# Phase-Change Memory from Molecular Tellurides

**DOI:** 10.1021/acsnano.3c10312

**Published:** 2023-12-20

**Authors:** Florian M. Schenk, Till Zellweger, Dhananjeya Kumaar, Darijan Bošković, Simon Wintersteller, Pavlo Solokha, Serena De Negri, Alexandros Emboras, Vanessa Wood, Maksym Yarema

**Affiliations:** † Chemistry and Materials Design Group, Institute for Electronics, Department of Information Technology and Electrical Engineering, ETH Zurich, Gloriastrasse 35, CH-8092 Zurich, Switzerland; ‡ Integrated Systems Laboratory, Department of Information Technology and Electrical Engineering, ETH Zurich, Gloriastrasse 35, CH-8092 Zurich, Switzerland; § Dipartimento di Chimica e Chimica Industriale, Università degli Studi di Genova, I-16146 Genova, Italy; ∥ Materials and Device Engineering Group, Institute for Electronics, Department of Information Technology and Electrical Engineering, ETH Zurich, Gloriastrasse 35, CH-8092 Zurich, Switzerland

**Keywords:** thin films, phase-change materials, tellurides, solution-based engineering, non-volatile memory devices

## Abstract

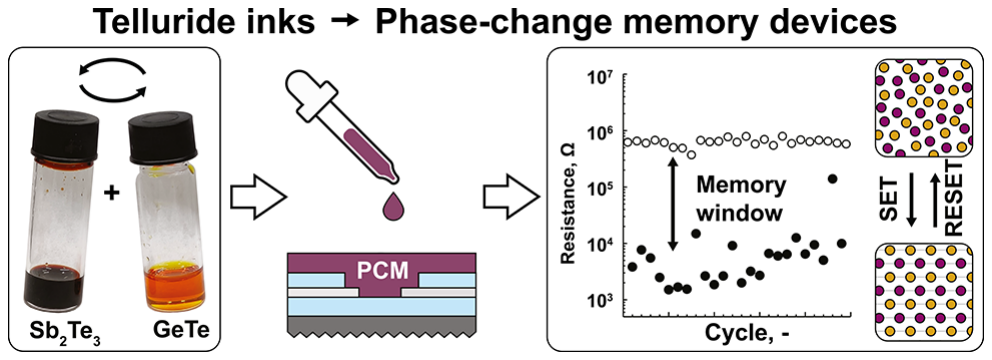

Phase-change memory (PCM) is an emerging memory technology based on the resistance contrast between the crystalline and amorphous states of a material. Further development and realization of PCM as a mainstream memory technology rely on innovative materials and inexpensive fabrication methods. Here, we propose a generalizable and scalable solution-processing approach to synthesize phase-change telluride inks in order to meet demands for high-throughput material screening, increased energy efficiency, and advanced device architectures. Bulk tellurides, such as Sb_2_Te_3_, GeTe, Sc_2_Te_3_, and TiTe_2_, are dissolved and purified to obtain inks of molecular metal telluride complexes. This allowed us to unlock a wide range of solution-processed ternary tellurides by the simple mixing of binary inks. We demonstrate accurate and quantitative composition control, including prototype materials (Ge–Sb–Te) and emerging rare-earth-metal telluride-doped materials (Sc–Sb–Te). Spin-coating and annealing convert ink formulations into high-quality, phase-pure telluride films with preferred orientation along the (00l) direction. Deposition engineering of liquid tellurides enables thickness-tunable films, infilling of nanoscale vias, and film preparation on flexible substrates. Finally, we demonstrate cyclable and non-volatile prototype memory devices, achieving performance indicators such as resistance contrast and low reset energy on par with state-of-the-art sputtered PCM layers.

The demand for data storage and data processing is increasing exponentially and is projected to reach approximately 175 zettabytes (1.75 × 10^11^ terabytes) in 2025.^1^ Additionally, the rapid development and expanding application of deep learning neural networks and large language models in everyday applications are further exacerbating the demand for data processing,^2^ driving the demand for alternative memory solutions. Phase-change memory (PCM) is among the most mature emerging memory technologies, offering faster read and write times, non-volatility at elevated temperatures, and multibit analog-type data storage potential.^3^ Phase-change memory is particularly suitable for in-memory computing, neuromorphic, and artificial intelligence applications.^4,5^ PCM stores information by using a stark contrast between the high-resistance amorphous and low-resistance crystalline phases of a material. To write the data, the PCM material is heated locally via optical or electrical pulses above the crystallization temperature (set process); amorphization via melt-quenching erases the data (reset process).^6^

Currently, PCM technology relies on prototypical ternary tellurides within the Ge–Sb–Te (GST) materials system, which have been intensively studied in relation to optical storage media.^7^ Importantly, key performance characteristics, such as data retention or programming speed, are a function of material composition,^8,9^ enabling application-specific, tailor-made memory. In particular, Ge-rich GST (Ge_*x*_Sb_2_Te_5_ with *x* > 2) exhibits higher crystallization temperatures up to 350 °C. Consequently, non-volatility (i*.*e., data retention for 10 years) can be achieved at elevated temperatures up to 185 °C,^10^ making Ge-rich GST materials a prime candidate for embedded memory in automotive applications and industry control integrative circuits.^10^ This, however, comes at a cost of slower programming speeds, typically larger than 1 μs. The prototypical stoichiometric Ge_2_Sb_2_Te_5_ (GST225) material demonstrates much faster switching speeds (down to 80 ns), but suffers from suboptimal data retention characteristics (10-year non-volatility at 85 °C).^10^ To overcome these trade-offs, transition-metal-doped Sb_2_Te_3_ materials, such as Sc–Sb–Te,^11^ Ti–Sb–Te,^12^ and Y–Sb–Te,^13^ recently have been proposed. These new PCM materials for instance feature higher crystallization temperatures, while reaching ultrafast switching speeds below 1 ns.^11^ Non-volatility combined with the performance of static RAM could drive the PCM technology closer toward a universal memory, meaning that PCM devices may replace single-handedly the storage-class memory and caches and even register in the architecture of future computers.^13^ The exploration of innovative PCM compositions, however, requires initial high-throughput screening of materials and devices, both computationally and experimentally.^11^ In order to permit this, simpler material preparation and device prototyping are necessary.

Solution-phase engineering of chalcogenides at ambient temperature and pressure provides a low-cost and scalable alternative,^14^ while enabling access to a wide range of compositions. Initially, hydrazine has been pioneered as a solvent for bulk chalcogenide powders such as CuInTe_2_^15^ and SnS.^16^ High-quality chalcogenide films were obtained via spin-coating and annealing, leading to functional devices such as thin-film transistors.^16^ In addition, phase-change Ge–Sb–Se thin films fabricated via the hydrazine route showed crystallization temperatures between 200 and 250 °C and laser-induced crystallization times below 1 μs. Despite this, no repeatable electrical switching has been reported for the hydrazine-processed materials.^17,18^ Colloidal nanocrystals represent another interesting class of liquid-fabricated PCM materials.^19−21^ Their unique size-dependent phase-change properties provide a roadmap for the ultimate scaling of PCM devices. Furthermore, colloidal nanocrystals have been employed for non-volatile reflective images as well as electrooptic and electronic memory devices.^21^ For example, colloidal GeTe nanoparticles can be electrically crystallized by applying 3 V/100 μs pulses and melt-quenched via 15 V/50 ns pulses for approximately 150 cycles featuring a 4-fold resistance contrast.^22^

Recently, amine-thiol cosolvents have emerged^23^ with the ability to dissolve a wide array of chalcogenides, oxides, and metals,^24^ at ambient pressure and temperature, using relatively benign chemicals. These solution-processed chalcogenides yield high-performance solar cells,^25^ resistive Ag_2_S-based memory,^26^ and thermoelectrics,^27^ yet no PCM device has been demonstrated to date. PCM is heavily reliant on telluride films, which remain underrepresented in the amine-thiol cosolvent formulations.^24^ Typically, tellurides are highly air- and moisture-sensitive compared to sulfides and selenides and have a tendency to form polytellurides (Te_*n*_^2–^) during dissolution.^28^ These polytelluride chains can aggregate to form structures of up to 50 nm,^28^ which is detrimental to the quality of telluride thin films. Postdissolution purification steps have been shown to improve the quality of sulfide^26^ and selenide inks.^27^ Recently, Jo et al.^28^ proposed a method to remove polytelluride byproducts via reduction with lithium triethylborohydride. This leads to improved film quality and thus high-performing thermoelectric devices.^28^

Here, we synthesize a range of PCM material inks by dissolving various bulk tellurides in an amine-thiol cosolvent and subsequent purification steps. We focus on the most promising PCM materials such as Sb_2_Te_3_, GeTe, Sc_2_Te_3_, or TiTe_2_ and use spin-coating deposition to achieve thin film telluride layers with tunable thickness, low surface roughness, and high crystallinity. We highlight the possibility of obtaining stoichiometric binary materials (e*.*g., Sb_2_Te_3_ or GeTe) as well as composition-tunable ternary materials, such as Ge_2_Sb_2_Te_5_ or Sc_0.2_Sb_2_Te_3_, by admixing telluride inks. This approach yields homogeneous thin films across a wide compositional range due to mixing on the molecular scale. We highlight the geometrical adaptability of liquid-phase processing, including the infilling of nanoscale vias and the deposition of films on flexible substrates. Finally, we demonstrate the fabrication and characterization of solution-based engineered memory devices. In particular, we quantify critical performance metrics such as *I*–*V* characteristics, switching parameters, resistance contrast, power consumption, and cyclability of functional memory devices, borne from molecular inks.

## Results and Discussion

### Solution Processing of Phase-Change Memory Tellurides

**Figure 1:**
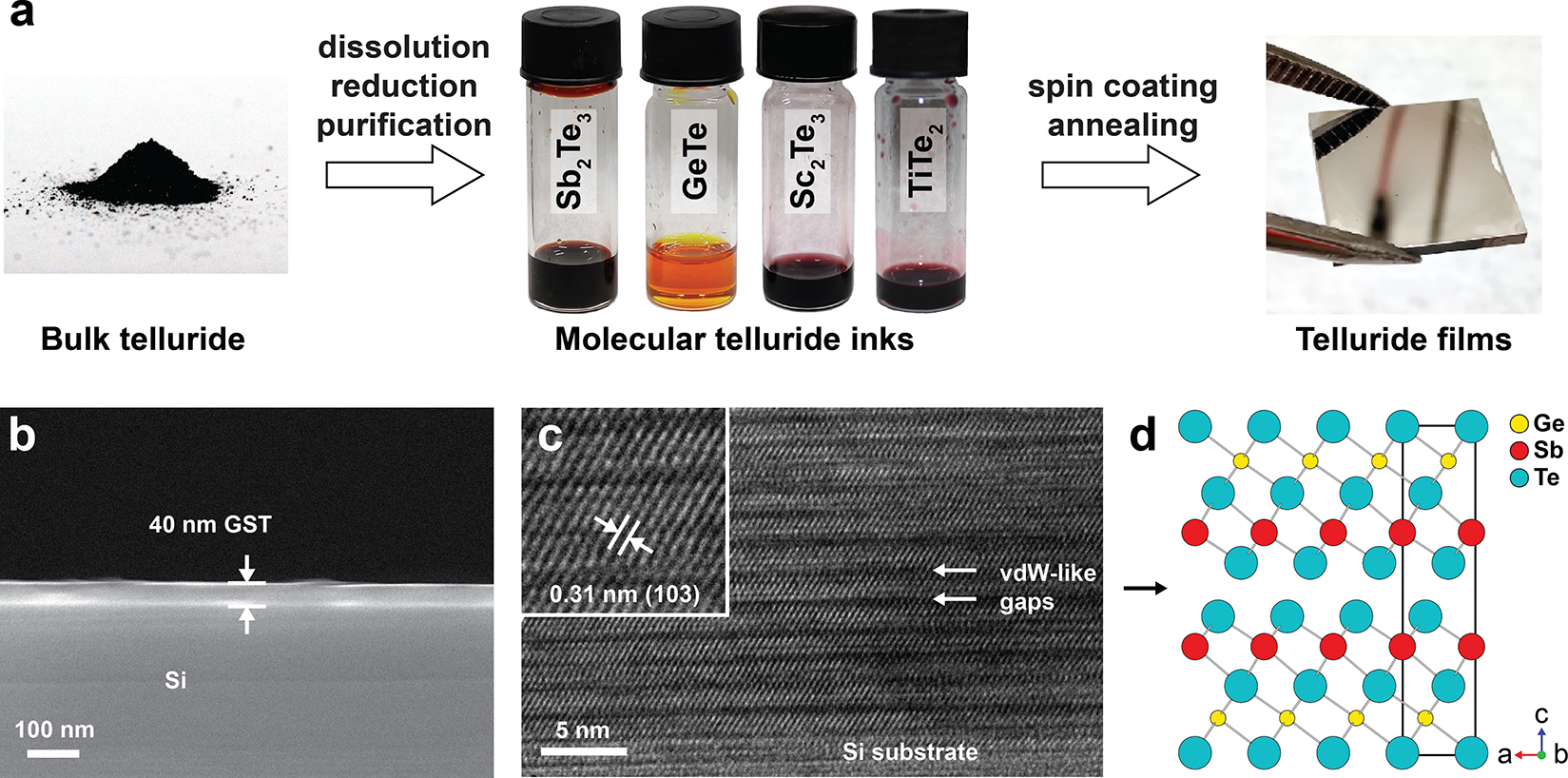
Preparing high-quality telluride thin films from molecular inks. (a) Schematics of the synthetic procedure, starting from bulk metal tellurides (Sb_2_Te_3_, GeTe, Sc_2_Te_3_, or TiTe_2_), and their conversion to molecular inks via dissolving in a cosolvent of ethylenediamine and 1,2-ethanedithiol, followed by spin-coating and annealing of pure inks or their mixtures. (b) Representative SEM image of 40 nm GST225 thin film with low roughness and uniform thickness. (c) HR-TEM image, highlighting high crystallinity and strong texture of a GST225 thin film in the (00l) direction. Arrows indicate van der Waals-like gaps parallel to the substrate. Inset measures lattice fringes with a distance of 0.31 nm, corresponding to (103) planes. (d) Crystal structure of rhombohedral Ge_2_Sb_2_Te_5_ (ICSD-188968), along the (010) direction.^34^

Figure 1a illustrates the process of converting bulk tellurides into telluride thin films via molecular inks. First, telluride powders are dissolved in the well-established cosolvent system of ethylenediamine and ethanedithiol.^24^ A rapid color change upon telluride addition indicates the formation of molecular chalcogenide complexes of Sb_2_Te_3_, GeTe, Sc_2_Te_3_, or TiTe_2_. The dissolution mechanism has been studied for sulfides and elemental sulfur.^29^ Specifically the thiolates, which form in an amine–thiol acid–base equilibrium, can break the metal–Te bonds via a series of base-catalyzed nucleophilic attacks.^30,31^ Ethylenediammonium cations then act as ligands, charge-balancing the anionic chalcogenide complexes. This process of dissolving metal tellurides, however, leads to the formation of polytelluride chains (Te_*n*_^2–^),^28^ which can form much larger structures (approximately 50 nm), compared to sub-1 nm metal chalcogenide complexes. Therefore, we follow a protocol developed by Jo et al. for Sb_2_Te_3_ inks to break polytelluride chains via reduction by lithium triethylborohydride.^28^ Subsequently, the crude molecular telluride solutions undergo purification via precipitation in acetonitrile and centrifugation, where the excess thiol is removed. Finally, after redissolving molecular complexes in ethylenediamine, excess elemental Te is removed by adding alkylphosphine, a well-known solvent for Te.^32^ Importantly, this synthetic procedure yields molecular telluride inks with the desired stoichiometry. Extending to other tellurides examined in this study, only minor adaptations are necessary. For example, initial dissolution time should be adjusted from 2 h for the case of Sb_2_Te_3_ to 15 h for Sc_2_Te_3_ and TiTe_2_, which can be associated with stronger Sc–Te and Ti–Te bonds compared to Sb–Te.^33^

In fabricating high-quality spin-coating layers, annealing is of particular importance.^35^ We employed thermogravimetric analysis (TGA) to estimate the optimal annealing temperature (Figure S1a). The majority of mass loss occurs between 150 and 300 °C, which can be attributed to the evaporation and thermal decomposition of the organic ligands. We therefore carry out the annealing of spin-coated layers at 350 °C and obtain uniform, organic-free, and specular reflective telluride thin films. The choice of annealing temperature takes into account the heating-ramp electrical resistance measurements (Figure S1b), which shows that the crystallization is complete around 250 °C for all investigated compositions. Furthermore, TGA allows a concentration estimation of as-synthesized Sb_2_Te_3_, GST225, and GeTe inks to be approximately 6 wt % by dividing the mass at 350 °C and the amount of ink used for the analysis (Figure S1a).

Figure 1b shows a cross-sectional SEM image of a typical GST225 thin film, highlighting its uniformity and laminar thickness of approximately 40 nm. Atomic force microscopy (Figure S2) further reveals a continuous coating with homogeneous granular features and the absence of pinholes, which are a reliability prerequisite for device fabrication. The surface is characteristic of low roughness with root-mean-square (RMS) surface roughness of just 6 nm, which is comparable to previously reported sulfide films.^26^ High-resolution TEM images reveal that our liquid-fabricated thin films have a high crystallinity (Figure 1c). The inset of Figure 1c shows lattice fringes with a distance of 0.31 nm, matching that of the (103) plane of GST225. Moreover, our telluride thin films attain a strongly preferred orientation in the (00l) direction, with the *c*-axis perpendicular to the Si substrate. The preferred orientation is also independent of the substrate (glass or silicon), and therefore it is likely driven by the thermodynamically lower surface energy of the exposed facet, which becomes even more significant for nanodimensional thin films.^36^ The Te-terminated surfaces of van der Waals-like gaps (indicated by arrows in Figure 1c and 1d) have particularly low surface energies,^37^ hence aligning parallel to the substrate interface. The strong texture in our telluride thin films is beneficial for PCM device performance, because cross-plane thermal conductivity is 60% lower compared to the in-plane direction.^38^ Reduced thermal conductivity across the film limits thermal dissipation and consequently improves energy consumption of PCM devices in crossbar configurations.^39^ Traditionally, the fabrication of highly textured PCM films requires specialized high-vacuum methods, such as molecular beam epitaxy^40^ or optimized sputtering conditions.^41^ Here, we provide a radically simpler approach through solution-processed chalcogenide thin films that spontaneously form preferred orientation upon annealing.^15,27^

### Ternary PCM Thin Films: From Ge–Sb–Te to Sc–Sb–Te

**Figure 2:**
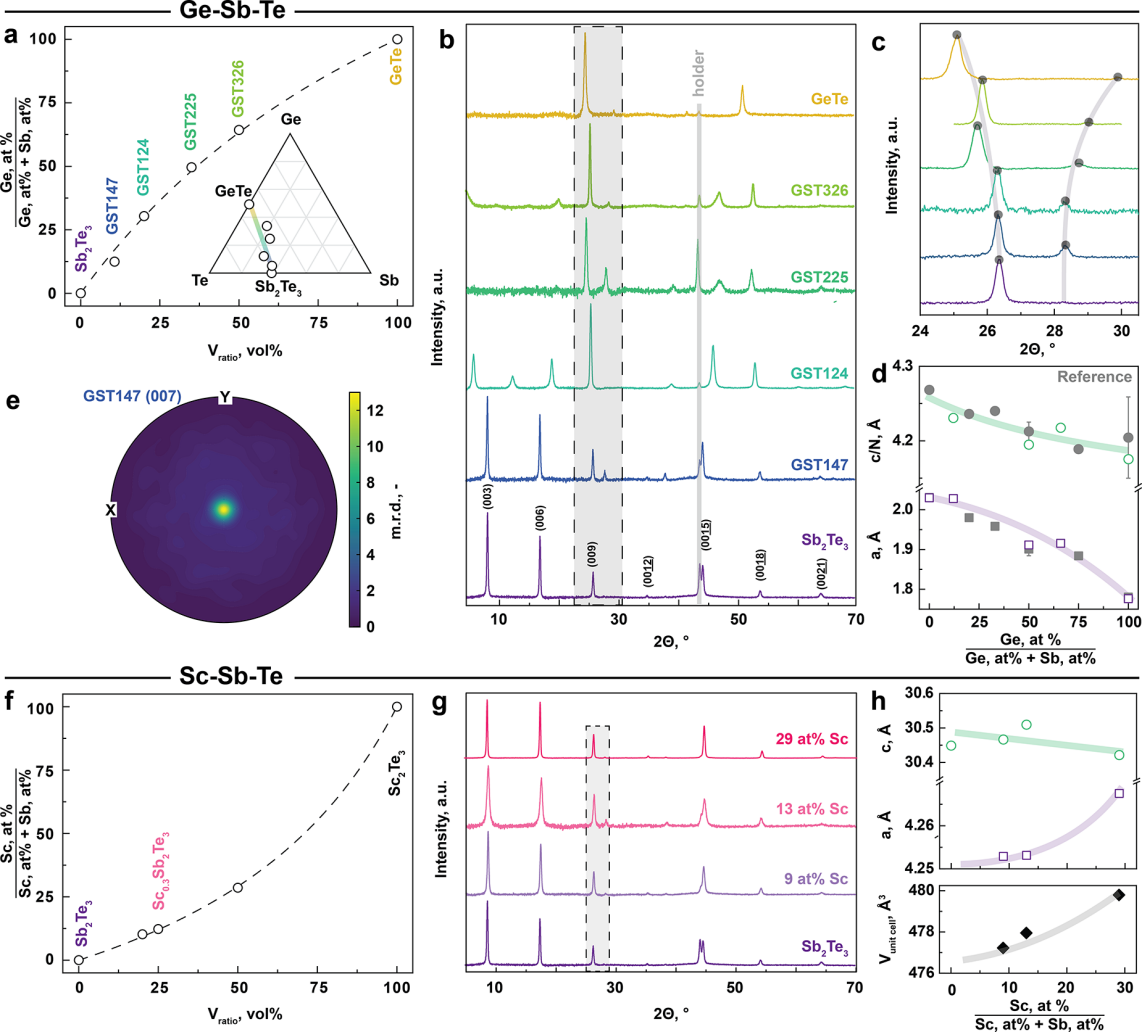
| Solution-processable ternary phase-change memory thin films. (a) Ge content in GST thin films as a function of the mixing ratio between GeTe and Sb_2_Te_3_ inks. Inset shows experimentally achieved compositions along the GeTe–Sb_2_Te_3_ pseudobinary line of the Ge–Sb–Te ternary phase diagram. (b) XRD patterns of the GST thin films with variable stoichiometries. Note that the peak at 44.1° originates from the stainless-steel holder. (c) A close-up view of XRD patterns, indicating a gradual Ge incorporation induces lattice contraction in the (00l) direction. (d) Lattice parameters *a* and *c*/*N* as a function of the Ge content (green circles and purple squares), plotted in comparison to the reference (gray circles and squares). Note that the lattice parameter *a* of Sb_2_Te_3_ could not be accurately extracted due to its strong texture. The lines are meant as a guide to the eye. (e) Pole figure of the (007) Bragg reflection of a GST147 thin film. The m.r.d. (multiples of random distribution) of 12.5 in the origin confirms the highly oriented nature of the film. (f) Sc content in Sc–Sb–Te thin films as a function of the mixing ratio between Sc_2_Te_3_ and Sb_2_Te_3_ inks. (g) XRD patterns of Sc-doped Sb_2_Te_3_ with variable Sc-doping amounts. (h) Lattice parameters *a* and *c* and the unit cell volume as a function of the Ge content (green circles and purple squares). The lines are meant as a guide to the eye.

We now focus on prototypical Ge–Sb–Te PCM materials as a case study. Simple admixing of GeTe and Sb_2_Te_3_ inks and subsequent annealing yields homogeneous ternary GST layers due to the mixing of metal chalcogenide complexes at the molecular scale, as shown by EDX elemental mapping in Figure S2. Figure 2a demonstrates accurate composition control, showing the Ge content of annealed GST thin films as a function of the volume ratio between the GeTe and Sb_2_Te_3_ inks. Importantly, our approach covers the whole range of stoichiometric GST compositions, including binary GeTe and Sb_2_Te_3_ materials and, for instance, the most prominent ternary telluride compositions, such as Ge_2_Sb_2_Te_5_, GeSb_2_Te_4_, or GeSb_4_Te_7_ (often denoted as GST225, GST124, or GST147 in the literature).^8^Figure S3 and Table S1 summarize the composition analysis, using energy-dispersive X-ray (EDX) spectroscopy.

Mixing the inks offers convenient and predictable composition control such that the Ge content gradually increases with the volume proportion (*V*_ratio_) in the mixed GeTe and Sb_2_Te_3_ inks. The composition function is monotonic and can be fitted using the following function:
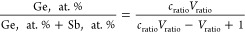
where *c*_ratio_ is the concentration ratio between GeTe and Sb_2_Te_3_ inks in at. % of the respective metal. If the inks are equally concentrated, *c*_ratio_ is equal to 1 and the function simplifies to a linear dependence with a slope of 1. If the GeTe or Sb_2_Te_3_ ink is more concentrated, then the composition function is concave or convex, respectively (Figure 2a). Throughout the study, the *c*_ratio_ needs to be determined for each pair of inks, ranging between 0.4 and 0.56 for our experiments.

The inset of Figure 2a shows the compositions within the Ge–Sb–Te ternary phase diagram and along the GeTe–Sb_2_Te_3_ tie-line. We note the influence of the stirring time on the final composition of the telluride inks (Figure S4). Longer dissolution of bulk tellurides leads to higher concentrations of sulfur in the resulting thin films, with proportionally smaller contents of Te. We associate it with strong binding of thiolate anions, particularly to GeTe molecular complexes. However, neither Te deficiency nor the presence of S should impede memory functionality, as demonstrated, for example, by the emergence of significantly Te-poor GST (e*.*g., Ge_4_Sb_6_Te_7_) compositions^42^ or by S-containing PCM materials.^43^ Eventually, sulfur-free GST thin films can also be achieved under optimal stirring times, except for binary GeTe, for which we consistently observe up to 10–15 at. % of S (Table S1).

For structural characterization, XRD patterns of Ge–Sb–Te thin films (annealed at 350 °C) are shown in Figure 2b. For all compositions, only reflections belonging to the respective thermodynamically stable trigonal phases can be observed, suggesting phase-pure and crystalline thin films. Evidently, telluride thin films have strong preferred orientation, as, for instance, the XRD of Sb_2_Te_3_ thin film contains only peaks corresponding to (00l) planes. The strong texture is characteristic for all compositions from Sb_2_Te_3_ up to GeTe, while the main (10l) Bragg reflection for GST compositions [(105) for Sb_2_Te_3_, (107) for GST124, or (103) for GST225] is only slightly noticeable. With increasing Ge content, the structure changes accordingly: The texture-related (00l) and main Bragg (10l) reflections move apart (Figure 2c), indicating lattice contraction due to the incorporation of smaller Ge atoms in the GST thin films. Figure 2d demonstrates a good agreement between our results and previous reference data, plotting the lattice parameter *a* and the average distance between two adjacent atomic layers *c*/*N* (where *N* is the number of layers per unit cell)^44^ as a function of the Ge content. Finally, we perform pole figure (PF) measurements to quantify the texture of the telluride thin films. As previously indicated by TEM and XRD (Figures 1c and 2b), liquid-borne telluride layers have a preferred orientation. Figure 2e shows a typical in-plane pole figure of a GST147 thin film, reaching a peak of multiples of random distribution (m.r.d.) value of 12 (m.r.d. of 1 means randomly distributed crystalline grains, i*.*e., ideal powder). Interestingly, regarding the degree of texture changes with the composition, binary Sb_2_Te_3_ thin films exhibit the strongest texture (m.r.d. of ca. 30) compared to Ge-containing GST147 and GST225 (m.r.d. values of 12 and 3.5, Figure S6). We argue that the lower surface energy for Te-terminated surfaces of van der Waals-like gaps can be a driving force for the preferential orientation. In support of this hypothesis, the slightly Te-deficient GST compositions have a lower degree of orientation, in contrast to stoichiometric Sb_2_Te_3_ (Table S1).

To demonstrate the universality of solution-processable tellurides, we extend our synthetic approach to Sc-doped Sb_2_Te_3_ thin films, which emerge as a highly promising emerging class of PCM materials.^11^ To obtain Sc-doped Sb_2_Te_3_, calculated amounts of Sc_2_Te_3_ ink are admixed to Sb_2_Te_3_ ink. Figure 2f shows the Sc content as a function of the mixing ratio between Sb_2_Te_3_ and Sc_2_Te_3_ inks, covering a wide range of Sc doping. We focus on achieving the most important PCM compositions, such as Sc_0.2_Sb_2_Te_3_ featuring sub-1 ns switching,^11^ and optimized Sc_0.3_Sb_2_Te_3_ with increased nucleation rates and improved stability of the amorphous phase.^45^ Notably, our liquid-processed Sc–Sb–Te compositions closely resemble the stoichiometry of the reported materials co-sputtered from Sc and Sb_2_Te_3_ (Table S2). XRD patterns (Figure 2g and Figure S5) show a trigonal Sb_2_Te_3_-type structure of Sc_*x*_Sb_2_Te_3_ thin films with up to 30 at. % of Sc content, with no other phases , such as cubic ScTe or trigonal Sc_2_Te_3_, present.^11^ It indicates high solubility of Sc_2_Te_3_ in Sb_2_Te_3_ material, related to the similar cationic sizes and crystal structures. Figure 2h shows the lattice constants *a* and *c* as a function of the Sc content. While *a* expands, *c* contracts slightly, resulting in an overall increase in the cell volume. This is consistent with the increased covalent radius of Sc compared to Sb (1.70 and 1.39 Å, respectively). Note that the increased density of defects, such as vacancies, may also play a role in the lattice dynamics for the Sc incorporation.

### Deposition Engineering of Molecular Tellurides

**Figure 3:**
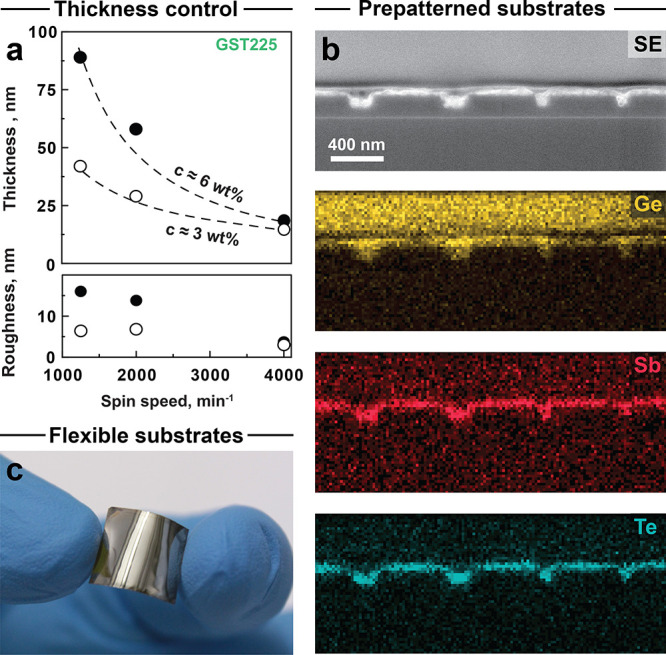
| Deposition engineering of GST225 telluride ink. (a) Film thickness and roughness as a function of the spin parameters, spinning speed, and ink concentration. (b) Nanoscale trenches (nominally, 200 and 100 nm), infilled with solution-processed GST225, as confirmed by cross-sectional SEM micrographs and EDX maps of Ge (in yellow), Sb (in red), and Te (in blue). (c) A GST225 film on a flexible polyimide substrate.

Spin-coating conformal telluride layers from the solution is a fast and effective process, which also further benefits the fabrication process. Namely, we can achieve accurate thickness control between 15 and 85 nm by varying the spinning speed (ω) and concentration of the ink (Figure 3a). The film thickness is proportional to the theory-predicted square root of the spinning speed (√ω^–1^).^46^ Comparing the 6 wt % (as-synthesized) and 3 wt % (diluted 1:1 (v/v) in ethylenediamine) GST225 inks, the concentration effect is almost linear at a low spinning speed of 1250 rpm, i*.*e., giving twice thinner layers for double-diluted inks. As expected, faster centrifugal forces of 4000 rpm alleviate the effect of the ink concentration (Figure 3a). Importantly, diluted inks result in smoother thin films with a roughness of just 6–8 nm, which allow precise engineering of ultrathin telluride thin films from the liquid phase.

Since solution-based deposition is not directly dependent on any particular substrate, we now test spin-coating of ternary telluride inks on prepatterned and flexible substrates. For the former, we designed a series of grooves with varying thickness (Figure S7a) and chose Si|SiO_2_ as a substrate. Molecular telluride inks show excellent infilling properties, even for the smallest sub-100 nm trenches (Figure 3b). Plasma-cleaned silica layers display particularly good wetting compatibility with molecular tellurides such that strong capillary forces drive the inks into the smallest gaps. Also, larger trenches up to 400 nm can be filled with the ink, leading to laminar continuous layers, reminiscent of ALD deposition (Figure S7b). With liquid processing, spatial confinement of PCM materials can be introduced in a singular processing step. Furthermore, our approach potentially enables the fabrication of high-density multilayer devices,^47^ where PCM inks should fill vias with higher aspect ratios.^48^ With regard to flexible substrates, we chose a polyimide (Kapton) thin film (Figure 3c). Due to its high thermal resistance, the same annealing protocol can be applied to polyimide/telluride stacks. It yields identical film quality compared to glass or silicon substrates, namely, specular reflective films and continuous layers. These telluride layers feature high film adhesion with no delamination upon bending (Figure 3c). Flexible polymer substrates can be interesting for embedded memory in flexible electronics or to improve energy consumption of memory devices due to the lower thermal conductivity of polyimide compared to Si.^49^

### Phase-Change Memory from Molecular Tellurides

**Figure 4:**
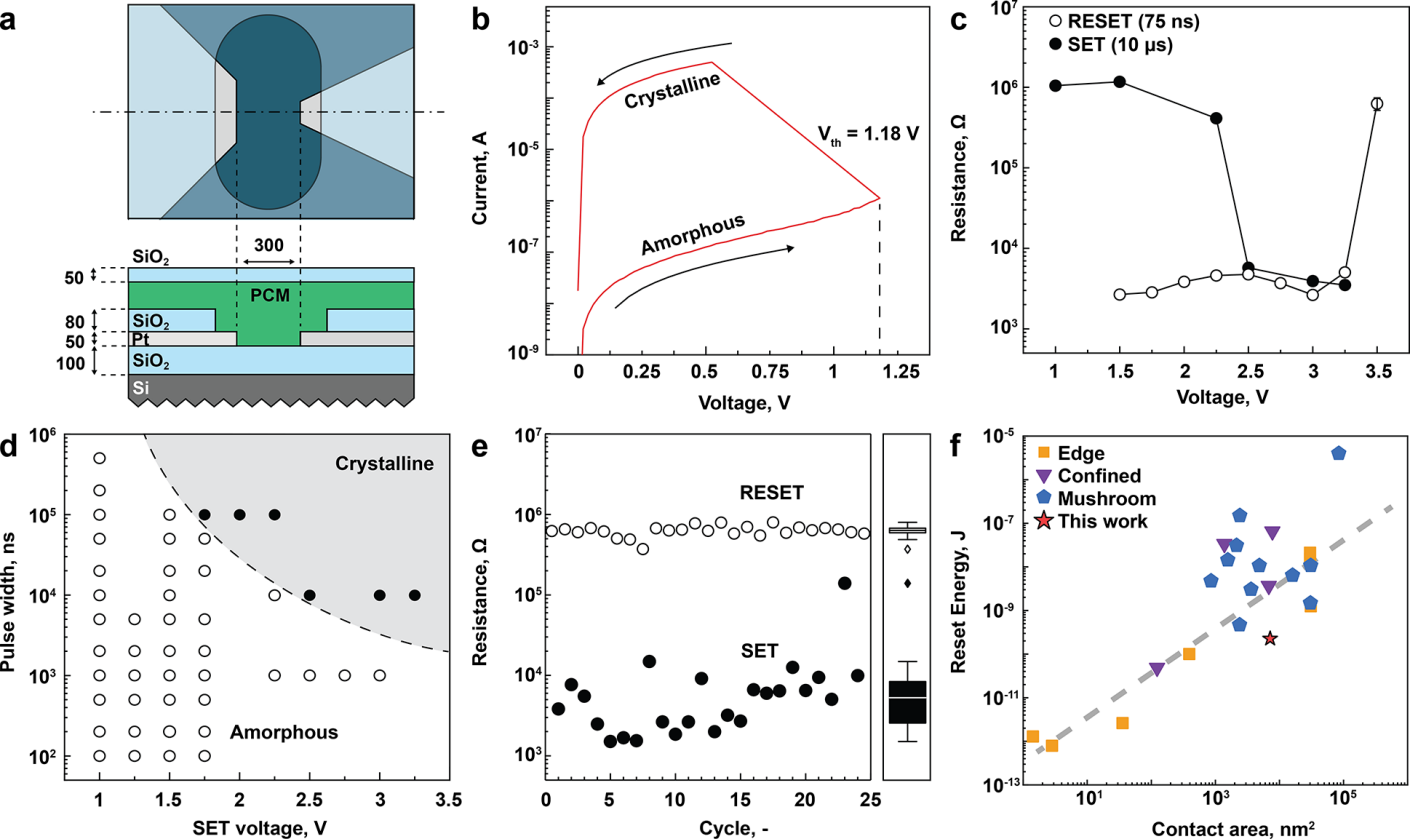
Phase-change memory device from molecular GST225 ink as a PCM layer. (a) Geometry of the prototype devices with the dimensions in nanometers. (b) *I*–*V* characterization of a melt-quenched device showing the characteristic “snapback” behavior upon crystallization at a threshold voltage (*V*_th_) of 1.18 V. (c) Cell resistance as a function of the SET or RESET pulse voltage with a duration of 10 μs (1 μs trailing edge) and 75 ns (2 ns trailing edge), respectively. (d) Parameter map of the SET pulse with varying pulse durations and voltages (100 ns trailing edge). Filled symbols denote conditions with successful crystallization (resistance decreases by at least a factor of 5). Open symbols depict no resistance change. (e) Cycling endurance of a memory cell with RESET (open symbols, 3.5 V, 75 ns, 2 ns trailing edge) and SET (filled symbols, 2.75 V, 10 μs, 1 μs trailing edge) pulses. The box-whisker diagram denotes median and 50% quantiles of the resistance after the SET and RESET pulses, respectively. (f) Comparison of RESET energy consumption with literature.^47^ The dashed line represents a linear fit of edge, confined, and mushroom device data.

After validating the excellent and controlled structural properties of telluride inks and thin films, we demonstrate functional phase-change memory materials in the form of prototype PCM devices in a planar configuration. Figure 4a illustrates the geometry of such a lateral device, which consists of two Pt electrodes, spaced some 300 nm apart. To increase confinement and to avoid sneak current paths, we cover the electrodes with a SiO_2_ layer and etch an oval area to locate the PCM device (top-view SEM image in Figure S8). We then deposit a GST225 ink via drop-casting and anneal the stack, creating a confined PCM device architecture. Finally, we add a thin silica capping layer for air and moisture protection. The initial resistance of the memory device is approximately 4 kΩ, which correspond to the crystalline phase of the GST225 film and agrees well with our van der Pauw measurements of bare spin-coated films and previous literature (Table S3). Figure 4b shows the *I*–*V* curve of a previously amorphized PCM device. With increasing voltage, the current increases exponentially, as expected for charge transport in amorphous semiconductors (Figure S9a).^50^ Then, at a threshold voltage (*V*_th_) of 1.18 V, the current rapidly increases, indicating crystallization of the GST225 layer due to local Joule heating. The *I*–*V* characteristics of several switching cycles are presented in Figure S9b, showing a *V*_th_ ranging from 0.8 to 2.0 V. We also demonstrate the memory switching via short electrical pulses (Figure 4c). To melt-quench (i.e., to amorphize) PCM material, we apply a short RESET pulse with a width of 75 ns and a 2 ns trailing edge. At voltages above 3.5 V, the device resistance increases by more than 2 orders of magnitude (up to approximately 1 MΩ), indicating the amorphization of the PCM material. To crystallize the PCM material, we apply a longer SET pulse of 10 μs in width and with a 1 μs trailing edge. The cell crystallizes at voltages above 2.5 V, as observed by the resistance drop back to around 4 kΩ. We report the RESET/SET resistance ratio of more than 2 orders of magnitude, which is comparable to state-of-the-art sputtered GST225 devices.^51^ Additional studies of pulse parameters are summarized in Figure 4d (100 ns trailing edge) and Figure S10 (1 μs trailing edge). As expected, applying smaller voltages (e*.*g., 1.75 V) may also result in crystallization SET switching of the memory device; however, it requires a longer pulse width of 100 μs. Overall, the map of SET parameters replicates a time–temperature–transformation (TTT) diagram of amorphous materials,^52^ further supporting that the resistivity contrast in our devices indeed comes from the phase transition of liquid-processed telluride thin films.

Our memory devices using a solution-processed PCM layer feature multiple cycles of memory switching with pronounced resistance contrast and energy consumption characteristics. Figure 4e demonstrates the resistance levels of a device during 25 full RESET/SET cycles. Overall, our PCM devices are non-volatile and switch reliably between the high-resistance and low-resistance states, while preserving a resistance window of over 2 orders of magnitude. The record device undergoes up to 200 switches (including parameter exploration explained above), demonstrating a cyclability comparable to other liquid-processed phase-change and resistive memory devices.^22,26^ Finally, we compare the reset energy of our prototype device to those of GST225 devices, fabricated with state-of-the-art sputtering methods (Figure 4f). For these, a general linear scaling law applies, and lower reset energies correlate with lower device dimensions (as quantified by the electrode area). Our liquid-processed GST device has a reset energy consumption of 229 pJ in an area of 7.200 nm^2^. This is almost 1 order of magnitude below GST devices with comparable dimensions, indicating reduced energy consumption of our PCM devices. Reset current and current density characteristics are also excellent: 0.875 mA and 12.2 MA cm^–2^, respectively. Further device miniaturization will enable liquid-borne PCM devices with superior ultralow power consumptions, increased cyclability, and increased memory density.

## Conclusions

In this work, we present high-performing phase-change memory devices from solution-processed amine–thiol inks. By dissolving inexpensive bulk tellurides and subsequent purification steps, stoichiometric inks for GeTe, Sb_2_Te_3_, Sc_2_Te_3_, and TiTe_2_ have been synthesized and spin-coated to obtain high-quality telluride thin films. By mixing the inks, we achieve accurate and predictable composition control over a wide range. As a universal approach, this enables access to important material classes for phase-change memory, from classical GST (Ge–Sb–Te) to emerging SST (Sc–Sb–Te) phase-change materials. We showcase the attractive abilities of liquid inks to tune the thin film thickness, infill vias in prepatterned substrates, and coat flexible substrates. Finally, functional materials have been demonstrated in the form of prototype memory devices. Important performance criteria, such as energy consumption and resistivity ratio, are comparable with devices made via conventional sputtering. With more sophisticated device fabrication and characterization, we anticipate significant improvement in cyclability, switching speed, and power consumption.

With the combined benefits of compositional tunability, deposition engineering, and functional phase-change devices, this process can pave the way for the low-cost solution-based fabrication of phase-change devices with high performance. For instance, the liquid ink PCM methodology can be readily extended by admixing non-stoichiometric dopants, such as In or Si, or mixed chalcogenides (GeTe_*x*_Se_1–*x*_ or Ge_*x*_Sn_1–*x*_Se) to tailor crystallization kinetics by several orders of magnitude.^9^ We also envision inkjet printing or layers patterned by optical lithography^53^ or direct light printing^54^ as inexpensive fabrication methods for phase-change memory. Furthermore, advanced computing architectures could be unlocked by infilling high-aspect-ratio devices or exploring the scaling limits by infilling memory cells in the sub-10 nm size regime. Finally, due to the conformal properties of liquid ink, optical elements such as waveguides could be coated effectively, enabling tunable photonic applications^55^ such as reconfigurable non-volatile displays,^56^ optical switches,^57^ or optical neuromorphic computing.^58^ Furthermore, telluride inks, as reported here, also present significant interest to other applications, such as thermoelectrics,^59,60^ photodetectors,^61^ solar cells,^62^ and ferroelectric memory.^63^ In light of this work, we hope to revive the dormant^17−19^ field of liquid-processed phase-change materials.

## Methods

### Materials

Antimony telluride (99.96%) and germanium telluride (99.999%) were purchased from ABCR, scandium metal (pieces, 99.99%) was from Smart-elements, titanium (lumps, 99.999%) from Merck, tellurium (broken ingots, 99.999%) and tri-*n*-octylphosphine (TOP, 97%) were from Strem, and ethylenediamine (≥99.5%), 1,2-ethanedithiol (>98%), toluene (anhydrous, 99.8%), Li(C_2_H_5_)_3_BH (1 M in THF), and acetonitrile (anhydrous, 99.8%) were from Sigma-Aldrich. All chemicals were used as received. All synthesis and processing steps were carried out under an inert atmosphere in a nitrogen-filled glovebox, unless noted otherwise.

### Synthesis of Sc_2_Te_3_ and TiTe_2_

Sc_2_Te_3_ was synthesized according to ref (64). In brief, Sc (0.285 g, 6.34 mmol) and Te (1.215 g, 9.52 mmol) were loaded in a Si_3_N_4_ ball-milling jar with stainless steel balls under an argon atmosphere. Ball milling was carried out on a Pulverisette 7 premium line (Fritsch) first with 8 mm balls for 2 h at 400 rpm and then with 1.5 mm balls for 16 h at 500 rpm, which yielded a fine dark gray powder. TiTe_2_ was synthesized via a solid-state chemistry route. Stoichiometric amounts of Ti dendrites and Te pieces were placed in a sealed glass ampule. The ampule was put inside a quartz tube and placed in a resistance furnace. The oven was inclined, and the tube protruded from one side to be able to rotate it from time to time during the synthesis. Slow heating to 280 °C was followed by an isothermal step overnight at this temperature. Subsequently, the sample was heated at 480 °C and kept for a while at this temperature: the appearance of some liquid was observed, and all of the material formed a cone at the bottom. Traces of sublimated Te were visible on the walls at the top of the vial. The ampule was then turned upside down to promote the evaporation of Te and incorporation into the sample. The temperature was set at 580 °C and kept at that temperature for another night. A dry, dark-crystalline material was obtained after this step. Figures S11 and S12 summarize the XRD and composition analyses (EDX) of synthesized tellurides.

### Synthesis of Molecular Telluride Inks

The molecular telluride inks were synthesized by dissolution in a cosolvent and subsequent purification, adapted from a previous report.^28^ Metal tellurides (175 mg Sb_2_Te_3_, 112 mg GeTe, 123 mg Sc_2_Te_3_, or 170 mg TiTe_2_) were added to ethylenediamine (5 mL) and 1,2-ethanedithiol (0.5 mL). The mixture was stirred for 2 h (Sb_2_Te_3_ and GeTe) or 15 h (Sc_2_Te_3_ and TiTe_2_) at 50 °C. Next, lithium triethylborohydride (0.5 mL) was added, and the solution stirred for another 15–20 min. Precipitation was carried out by separating the solution in two centrifuge tubes, adding acetonitrile (42 mL) to each, and centrifuging at 8000 rpm for 8 min. The supernatant was discarded, and the pellet was redissolved in ethylenediamine (a total of 1 mL). After filtration through a 0.2 μm PTFE filter, TOP (0.25 mL) was added, and the solution was shaken for 90 s. For Sc_2_Te_3_, a larger volume of TOP (0.5 mL) was added. In the next step, toluene (2 mL) was added and, after shaking for another 90 s, the upper phase was removed, after which ethylenediamine (0.15 mL) was added. The toluene washing step was repeated twice. Finally, the inks were filtered through a 0.2 μm PTFE filter. The inks were stable for at least several months under a nitrogen atmosphere.

### Film Deposition

Substrates (glass or silicon with a native oxide layer) were cleaned twice by ultrasonication in Mucasol (10 vol %), twice in deionized water, then in acetone, and finally by a submerging in boiling isopropanol (5 min for each step), followed by oxygen plasma cleaning. Polyimide substrates were rinsed with isopropanol. The spin-coating protocol consists of a prespin step at 400 rpm for 5 s, followed by a spinning step for 60 s at the final speed between 1250 and 4000 rpm. An acceleration time of 0.5 s was used. The films were placed on a temperature-controlled hot plate, dried at 70 °C for 10 min, and then annealed at 350 °C for 20 min with a ramp of 5 °C·min^–1^. The as-deposited thin film is characteristic of an amorphous structure (Figure S13).

### Materials Characterization

X-ray diffraction was carried out on a Rigaku SmartLab 9 kW system, equipped with a rotating Cu anode (45 kV and 200 mA) and a 2D solid-state detector (HyPix-3000 SL). Films were spun at 2000 rpm on a glass substrate. Note that the spin-coating conditions influence the thickness of thin films and the degree of their texture (Figure S14). Rietveld analysis was carried out using the software Match! (Crystal Impact). Pole figures were recorded in the in-plane geometry. After absorption correction and background subtraction, the experimental pole figures were used to calculate the orientation distribution functions using the MTEX toolbox in MATLAB. From these, normalized pole figures were obtained. Atomic force microscopy (AFM) was carried out on an Agilent 5500 instrument in tapping mode under ambient conditions. The root-mean-square (RMS) roughness was calculated after subtraction of a polynomial background using the Gwyddion software. The thickness was determined by measuring the profile of the film scratched by a razor blade. TGA measurements were performed under a nitrogen purge flow of 40 mL/min using a TGA Q50 instrument (TA Instruments). The inks were filled into alumina crucibles and then placed onto a platinum pan and heated at a rate of 5 °C/min from room temperature to 100 °C, kept there for 30 min, and then heated to 450 °C. Data sets were normalized relative to the sample mass in order to calculate the percentage mass loss. Secondary electron microscopy imaging was carried out on an S-4800 FE-SEM (Hitachi). Energy-dispersive X-ray spectroscopy was carried out on an FEI Quanta 200F equipped with an Octane Super EDX detector and operated at 30 kV. Elemental quantification was carried out using the EDAX Apex software on 40 × 40 μm sections across the film. Accuracy was confirmed by using commercial Sb_2_Te_3_ and GeTe powders. Cross-sectional imaging and preparation of the TEM lamella were performed on a Helios 5 UX FIB-SEM, Thermo Fischer Scientific. Cross sections and lamella specimens were prepared using a gallium focused ion beam (Ga FIB). The TEM lamella was prepared with carbon as the protection layer, and the final polishing of the lamella was performed at 5 and 2 kV. PCM materials were imaged using a high-angle annular dark-field (HAADF) detector on a Helios 5 UX. EDX was measured with an Oxford Ultim Max 100 mm^2^ detector. HR-TEM of lamella specimens was carried out on a Talos F200X instrument operated at 200 kV.

### Device Fabrication

Prototype devices were fabricated on a Si substrate with 100 nm of thermally deposited SiO_2_. Electrodes were fabricated via electron beam lithography, electron beam evaporation of platinum, and a subsequent lift-off in hot DMSO. Subsequently, 80 nm SiO_2_ was deposited on the sample using plasma-enhanced chemical vapor deposition (PECVD). To etch nanosized holes in the switching area of the devices and to free the pads for electrical contacts, electron beam lithography with a PMMA mask followed by an RIE dry etch was performed. GST225 ink was drop-cast locally around the device area to fill the nanosized holes. After annealing, the material was capped with 50 nm SiO_2_.

### Electrical Characterization

Sheet resistance was measured using the van der Pauw method with four terminal electrodes connected to a Keithley 2400 SMU. Four measurements were taken per film. Temperature-dependent resistance measurements were carried out using electrodes connected to microneedle positioners. The films were electrically characterized inside the glovebox right after spin coating and annealing for 10 min at 70 °C, and measurements were taken every five seconds during a linear temperature sweep. Pulse measurements were performed by applying voltage pulses with variable width as well as variable leading and trailing edges by the Keysight 33600A arbitrary waveform generator and measuring the in- and output voltage of the device under test using the RTE 1000 oscilloscope from Rhode & Schwarz. A series resistance of 1 kΩ was used to limit the maximal current through the device. The RV sweeps and DC resistance measurements were carried out using the Keysight B2902A precision source. For the first formation cycle, a 6.25 V (50 ns) pulse is required for successful reset switching. Reset energy was calculated using the equation  with *R*_set_ being the cell resistance before the reset pulse of *V*_reset_ and *t*_reset_ (the nominal reset pulse amplitude and width, respectively) was applied. This provides a conservative upper limit for energy consumption.

## Supporting Information Available

Thermogravimetric analysis, X-ray diffraction patterns, composition analysis via energy-dispersive X-ray spectroscopy, microscopy, and electrical characterization (PDF)
