# Supplementary material for: Phase-Change Memory from Molecular Tellurides
Source: ACS Nano. 2023 Dec 20;18(1):1063–72. doi: 10.1021/acsnano.3c10312 (PMC10786157; doi:10.1021/acsnano.3c10312)
Supplement: Supplementary file 1 — nn3c10312_si_001.pdf [file nn3c10312_si_001.pdf]

Supporting Information

Phase-Change Memory from Molecular Tellurides

*Florian M. Schenk,[1] Till Zellweger,[2] Dhananjeya Kumaar,[1] Darijan Bošković,[1] Simon Wintersteller,[1] Pavlo Solokha,[3] Serena De Negri,[3] Alexandros Emboras,[2] Vanessa Wood,[4] and Maksym Yarema[1]**

[1]Chemistry and Materials Design Group, Institute for Electronics, Department of Information Technology and Electrical Engineering, ETH Zurich, Gloriastrasse 35, CH-8092 Zurich, Switzerland

[2]Integrated Systems Laboratory, Department of Information Technology and Electrical Engineering, ETH Zurich, Gloriastrasse 35, CH-8092 Zurich, Switzerland

[3]Dipartimento di Chimica e Chimica Industriale, Università degli Studi di Genova, I-16146 Genova, Italy

[4]Materials and Device Engineering Group, Institute for Electronics, Department of Information Technology and Electrical Engineering, ETH Zurich, Gloriastrasse 35, CH-8092 Zurich, Switzerland

* Correspondence to yaremam@ethz.ch

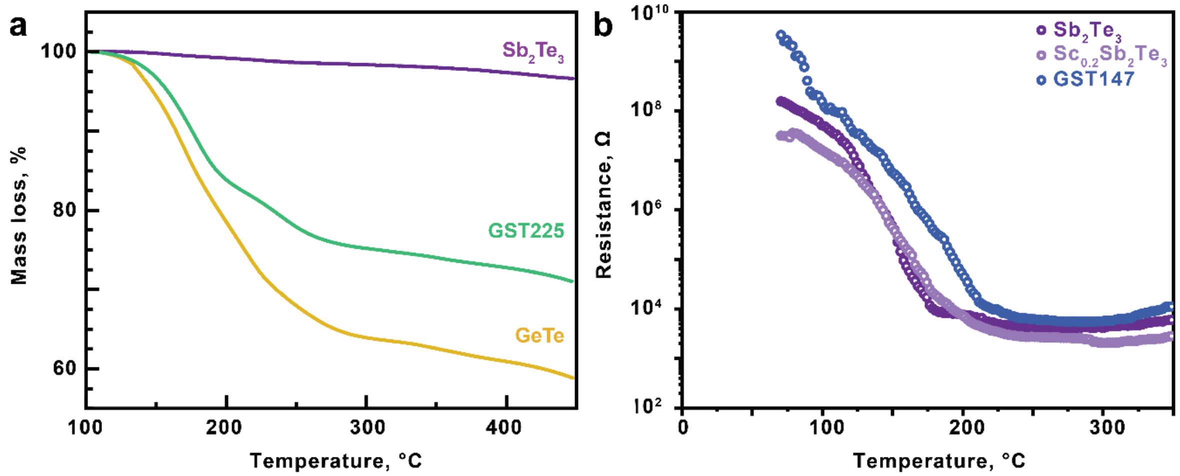

Figure S1. a) Thermogravimetric analysis of Sb_2Te_3, GeTe and GST225 inks at a ramp rate of 5 K min^{-1} and under N_2 flow. b) Temperature-ramp resistance measurements of Sb_2Te_3, $Sc_{0.2}Sb_2Te_3$ and GST147 thin films via a two-probe setup. The heating rate was 5 K/min. Note that during the heating ramp, several processes occur sequentially and at different temperatures, such as evaporation of the ethylenediamine (bp = 116 °C), evaporation of the ethanedithiol (bp = 146 °C), loss of bound organic ligands at higher temperatures, and finally, loss of Te at > 400 °C.

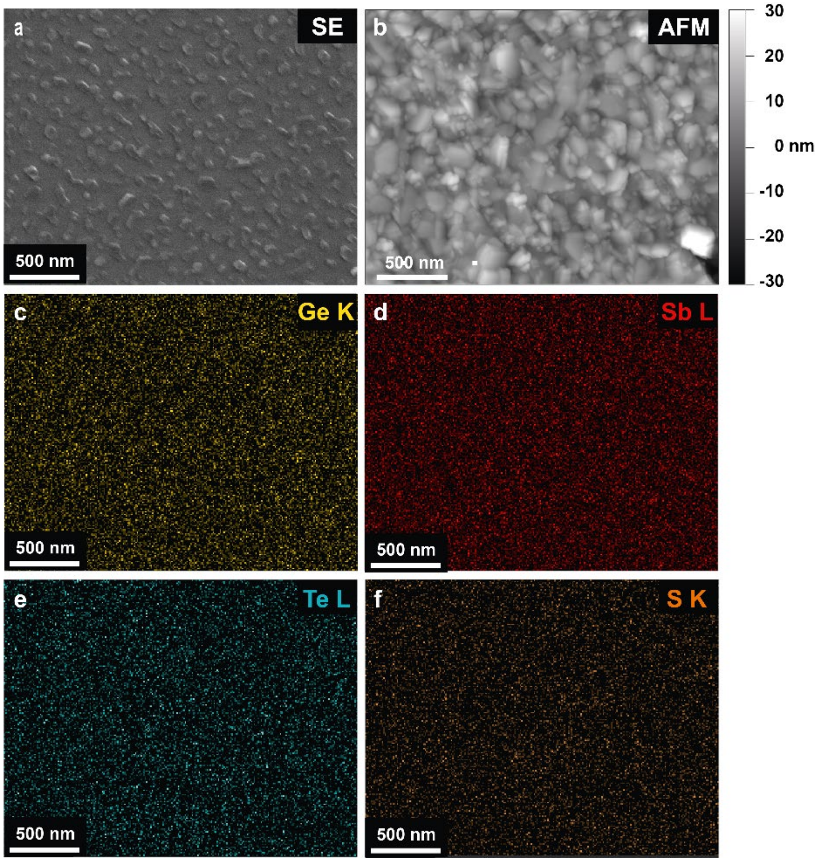

Figure S2. a) Secondary electron micrograph, b) atomic-force microscopy image and c-f) EDX composition mapping of the thin film with nominal composition of GST225. EDX quantification of the film resulted in 23.6 at% Ge, 25.5 at% Sb, 47.3 at% Te, and 3.6 at% S, showing that elements are homogenously distributed within the film.

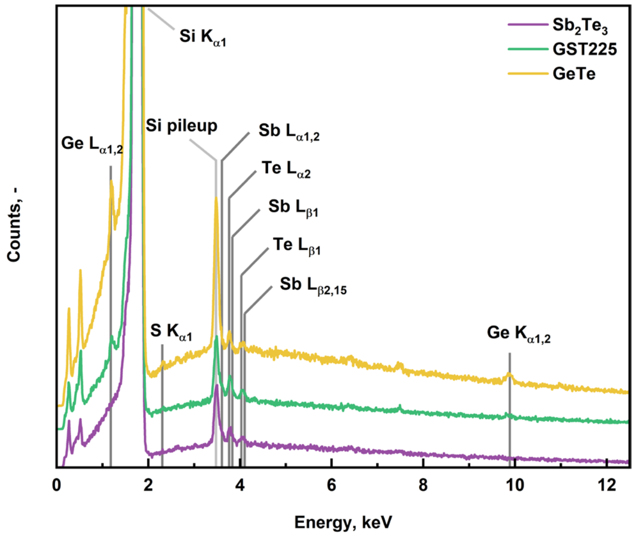

Figure S3. Energy-dispersive X-ray spectra of GeTe, GST225, Sb_2Te_3 thin-films indicating the presence or absence of Ge, Sb, and Te elements in PCM layers. The pile-up peak at 3.48 keV originates from simultaneous absorption of two Si Ka x-rays (2 × 1.74 eV = 3.48 keV) due to the strong Si signal from the substrate.

Table S1. Elemental composition of Ge-Sb-Te thin films as quantified by EDX spectroscopy.

Ink mixing ratio GeTe-Sb_2Te_3, % (v/v)	Ge, at%	Sb, at%	Te, at%	S, at%	$\frac{\text{Ge, at\%}}{\text{Ge, at\%+Sb, at\%}}$, %
0	0.0	37.4	59.6	3.0	0
11	5.2	36.2	58.6	0.0	12.5
20	12.1	27.7	60.3	0.0	30.4
35	23.6	24.0	48.0	4.4	49.6
50	33.3	18.5	46.9	1.4	64.3
100	43.6	0.0	44.6	11.8	100

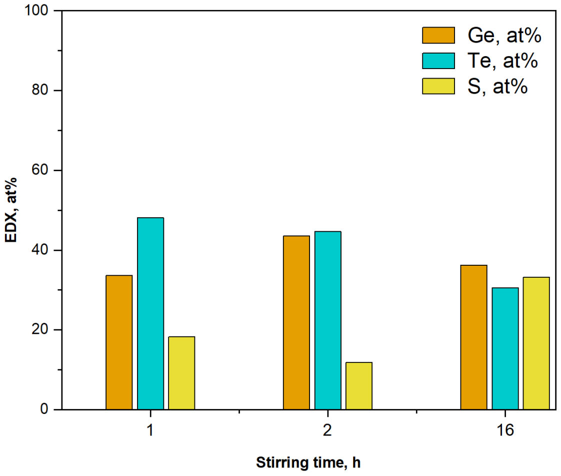

Figure S4. Composition of GeTe thin films in relation to the total stirring time of bulk GeTe in the amine-thiol co-solvent mixture. The elemental ratios were determined by EDX spectroscopy. For shorter stirring time (1 h), the films are tellurium-rich despite intense purification protocol with alkylphosphine. Longer stirring (> 4 h) leads to proportionally higher sulfur contents.

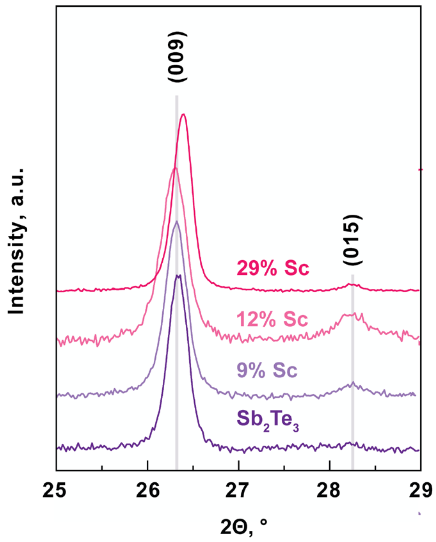

Figure S5. Zoom-in on the XRD pattern of Sc-Sb-Te from Figure 2g.

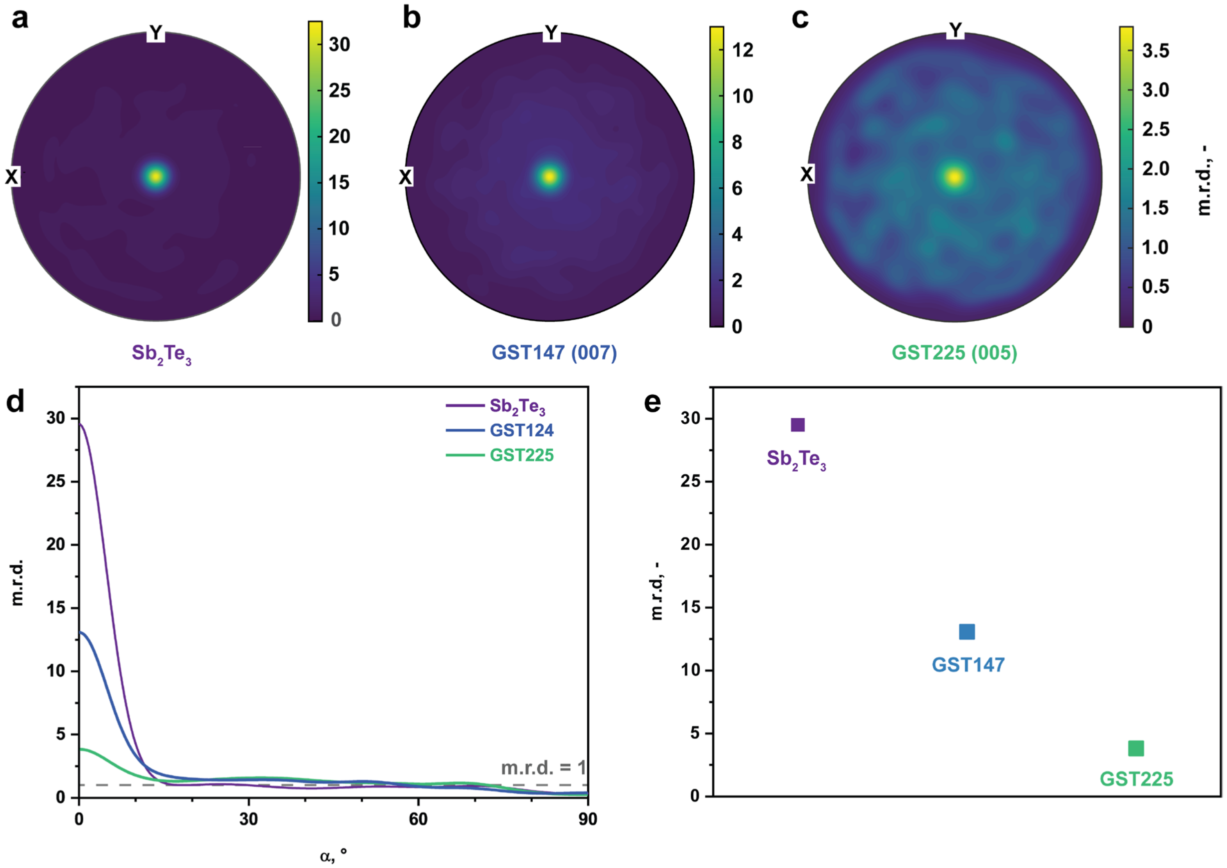

Figure S6. In-plane pole figures of a) Sb_2Te_3, b) GST147 and c) GST225 thin films. d) Radial integration yields the multiples of random distribution (m.r.d.) as a function of the tilt angle α. Higher m.r.d. values indicate stronger preferred orientation of thin films. e) The degree of texture (m.r.d. value at the origin) as a function of increasing Ge content in thin films.

Table S2. Elemental content of Sc-Sb-Te thin films as quantified by EDX spectroscopy.

Ink mixing ratio Sc_2Te_3-Sb_2Te_3, % (v/v)	Sc, at%	Sb, at%	Te, at%	S, at%	$\frac{\text{Sc, at\%}}{\text{Sc, at\%+Sb, at\%}}$, %
0	0.0	37.4	59.6	3.0	0.0
20	4.4	38.8	56.8	0.0	10.2
25	5.4	38.4	56.2	0.0	12.3
50	12.9	32.3	54.8	0.0	28.6
100	32.5	0.0	67.5	0.0	100.0

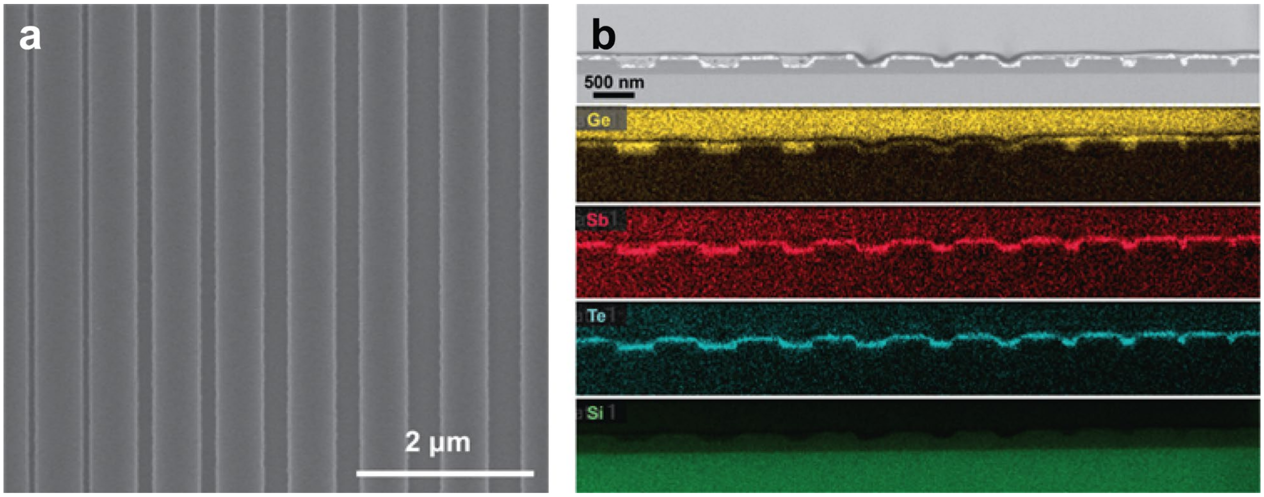

Figure S7. Deposition of GST225 molecular inks on pre-patterned Si|SiO_2 substrates. a) Top-view SEM image of the patterned trench structures before the ink deposition. b) Full cross-sectional SEM image and SEM-EDX maps of the infilled trenches, as shown in Figure 3b. Ge in yellow, Sb in red, Te is blue, Si in green.

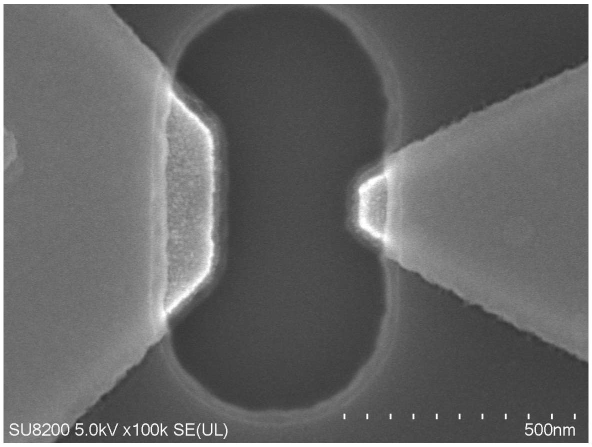

Figure S8. Top-view SEM image of a PCM device with a gap of 300 nm prior to infilling with telluride ink.

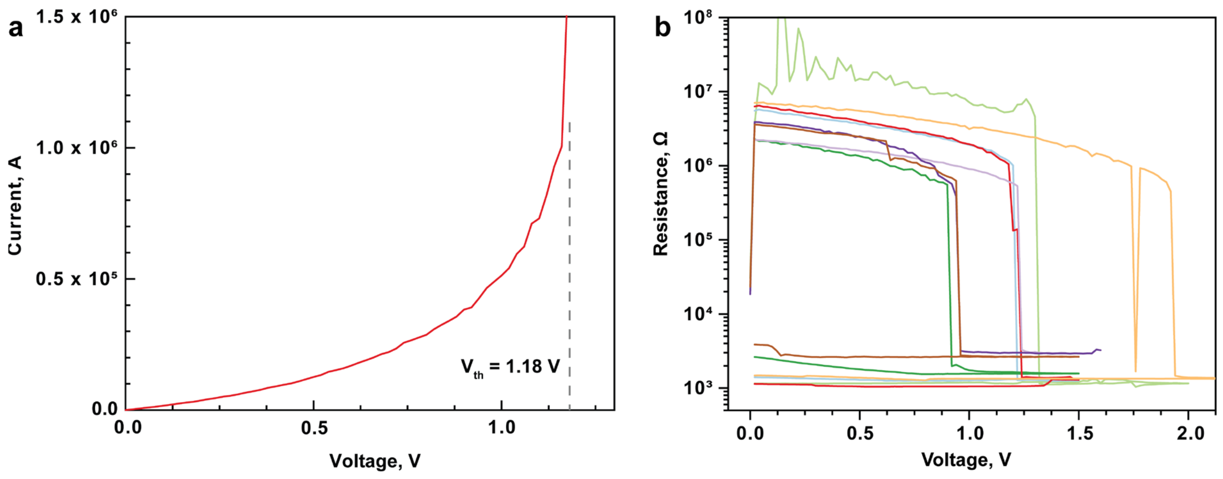

Figure S9. Extended I-V characterization of the GST225 device. a) I-V sweep of a melt-quenched device plotted linearly. This demonstrates the exponential increase of current with increased voltage. b) I-V sweep of melt-quenched devices exhibiting the "snapback" behaviour for 9 independent measurements. The threshold voltage lies between 0.9 and 1.9 V.

Table S3. Sheet resistance of crystalline Sb_2Te_3, $Sc_{0.2}Sb_2Te_3$ and GST147 as determined by the van-der-Pauw method along with the literature values, given as comparison.

Material	Deposition method	Film thickness, nm	Sheet resistance, Ω/□	Resistivity, Ω·m	Ref.
Sb_2Te_3	Sputtering	200	1 200	$2.4\cdot10^{-4}$	[1]
- // -	Sputtering	120	1 700	$2.0\cdot10^{-4}$	[2]
- // -	Spin coating	220	100	$2.2\cdot10^{-5}$	[3]
**- // -**	**Spin coating**	**60**	**4 000**	$\mathbf{9\cdot10^{-4}}$	**This work**
$Sc_{0.2}Sb_2Te_3$	Sputtering	300	1 000	$3\cdot10^{-4}$	[4]
- // -	Sputtering	300	2 800	$8\cdot10^{-4}$	[5]
**- // -**	**Spin coating**	**30**	**300**	$\mathbf{3.5\cdot10^{-5}}$	**This work**
$Ge_1Sb_4Te_7$	Sputtering	95	-	$1\cdot10^{-3}$	[6]
**- // -**	**Spin coating**	**10**	**7 500**	$\mathbf{1.8\cdot10^{-4}}$	**This work**

[1] Zhu, M.; Wu, L.; Rao, F.; Song, Z.; Ren, K.; Ji, X.; Song, S.; Yao, D.; Feng, S. Uniform Ti-Doped Sb_2Te_3 Materials for High-Speed Phase Change Memory Applications. *Appl. Phys. Lett.* **2014**, *104 (5)*, 53119.
[2] Sun, L.; Chen, Y.; Mao, Y.; Meng, Y.; Gu, C.; Shen, X.; Xu, T. Enhancement of Thermal Stability by Calcium Doping in Sb_2Te_3 for Ultrastable Phase-Change Memory. *J. Non. Cryst. Solids* **2022**, *577*, 121327.
[3] Jo, S.; Park, S. H.; Shin, H.; Oh, I.; Heo, S. H.; Ban, H. W.; Jeong, H.; Kim, F.; Choo, S.; Gu, D. H.; Baek, S.; Cho, S.; Kim, J. S.; Kim, B. S.; Lee, J. E.; Song, S.; Yoo, J. W.; Song, J. Y.; Son, J. S. Soluble Telluride-Based Molecular Precursor for Solution-Processed High-Performance Thermoelectrics. *ACS Appl. Energy Mater.* **2019**, *2 (7)*, 4582.
[4] Ding, K.; Chen, B.; Chen, Y.; Wang, J.; Shen, X.; Rao, F. Recipe for Ultrafast and Persistent Phase-Change Memory Materials. *NPG Asia Mater.* **2020**, *12 (1)*, 1.
[5] Rao, F.; Ding, K.; Zhou, Y.; Zheng, Y.; Xia, M.; Lv, S.; Song, Z.; Feng, S.; Ronneberger, I.; Mazzarello, R.; Zhang, W.; Ma, E. Reducing the Stochasticity of Crystal Nucleation to Enable Subnanosecond Memory Writing. *Science* **2017**, *358 (6369)*, 1423.
[6] Sahu, S.; Pandey, S. K.; Manivannan, A.; Deshpande, U. P.; Sathe, V. G.; Reddy, V. R.; Sevi, M. Direct Evidence for Phase Transition in Thin $Ge_1Sb_4Te_7$ Films Using in Situ UV–Vis–NIR Spectroscopy and Raman Scattering Studies. Phys. *Status Solidi B Basic Res.* **2016**, *253 (6)*, 1069.

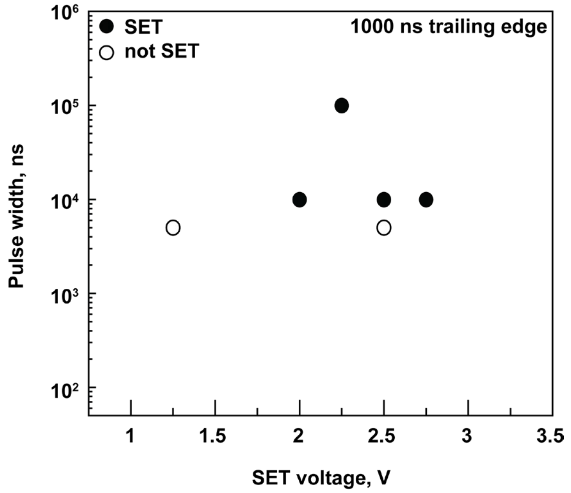

Figure S10. Parameter map of the SET pulse with a trailing edge of 1000 ns. Filled symbols denote conditions with successful crystallization (resistance decrease by at least a factor of 5). Open symbols depict no resistance change.

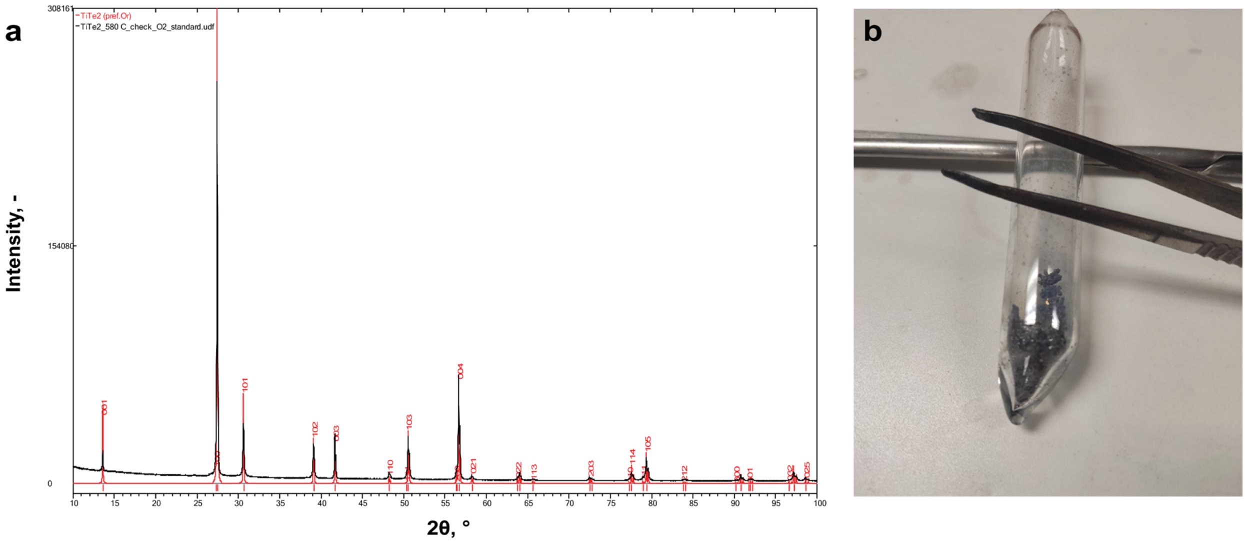

Figure S11. Solid-state synthesis of $TiTe_2$. a) XRD of $TiTe_2$, prepared via solid-state synthesis method, b) Image of the ampoule after synthesis

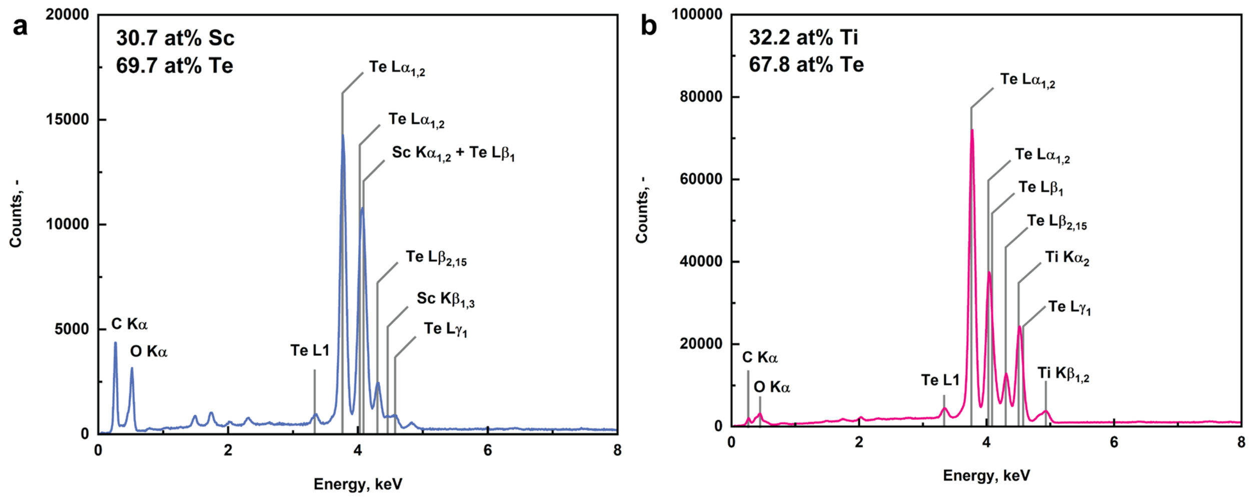

Figure S12. EDX spectra of Sc_2Te_3 and $TiTe_2$ powders and their corresponding quantification results.

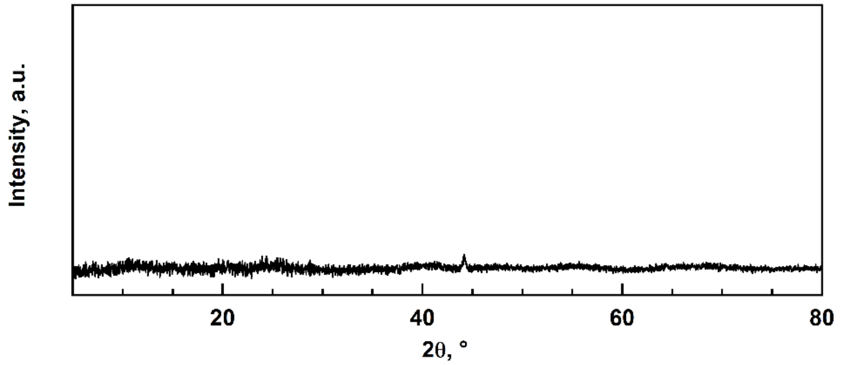

Figure S13. XRD pattern of a GST225 film, dried at 70 °C for 10 min, prior to annealing. The film is nominally amorphous, as only a peak at 44.1 °C originating from the holder can be observed.

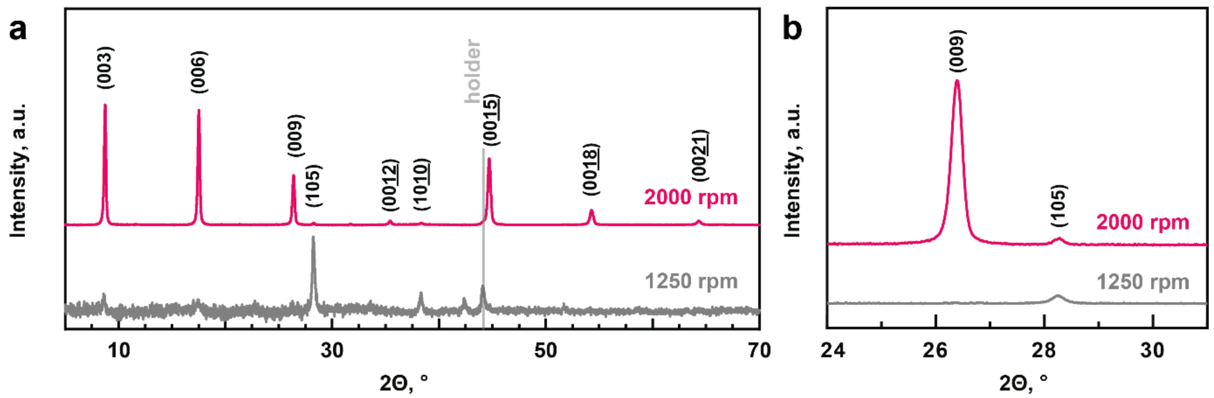

Figure S14. XRD pattern of Sb_2Te_3 doped with 29 at% Sc, prepared with different spin-coating conditions. Slower spin speed (1250 rpm) leads to thicker films with less texture, compared to faster speeds (2000 rpm), as evidenced by weaker (00l) and more pronounced (105) reflections.
